# 100 years since the publication of the suramin formula

**DOI:** 10.1007/s00436-023-08027-7

**Published:** 2023-12-07

**Authors:** Dietmar Steverding, Linda Troeberg

**Affiliations:** Bob Champion Research and Education Building, Norwich Medical School, University of East Anglia, Norwich Research Park, Rosalind Franklin Road, Norwich, NR4 7UQ UK

**Keywords:** Suramin, Sleeping sickness, Political instrumentalization, Colonial revisionism

## Abstract

Suramin was the first drug developed using the approach of medicinal chemistry by the German Bayer company in the 1910s for the treatment of human African sleeping sickness caused by the two subspecies *Trypanosoma brucei gambiense* and *Trypanosoma brucei rhodesienese*. However, the drug was politically instrumentalized by the German government in the 1920s in an attempt to regain possession of its former African colonies lost after the First World War. For this reason, the formula of suramin was kept secret for more than 10 years. Eventually, the French pharmacist Ernest Fourneau uncovered the chemical structure of suramin by reverse engineering and published the formula of the drug in 1924. During the Nazi period, suramin became the subject of colonial revisionism, and the development of the drug was portrayed in books and films to promote national socialist propaganda. Ever since its discovery, suramin has also been tested for bioactivity against numerous other infections and diseases. However, sleeping sickness caused by *Trypanosoma brucei rhodesiense* is the only human disease for which treatment with suramin is currently approved.

## Introduction

Suramin is a polysulfonated naphthylurea developed in the 1910s by the German Bayer company (then known as Farbenfabriken vorm. Friedr. Bayer & Co. (Bayer 2023)) for the treatment of human African trypanosomiasis or sleeping sickness caused by the two subspecies *Trypanosoma brucei gambiense* and *Trypanosoma brucei rhodesiense*^1^ (Low and Manson-Bahr 1923; Steverding 2008). The drug (under the name naganol) was also investigated for the treatment of animal trypanosomiasis (Van Saceghem 1925; Hetzel 1942). Suramin was found to be much more effective against trypanosomes than any previously used remedies and had fewer side effects. At the beginning of the twentieth century, sleeping sickness was a big problem in Africa, and the development of suramin was a huge breakthrough in the therapy of the disease (Steverding 2010). The drug is still used today for the treatment of the early stage of East African sleeping sickness caused by *T. b. rhodesiense* (Steverding 2017) (*T. brucei* SRA+ (Steverding and Tyler 2021)) and is the standard treatment of surra (*Trypanosoma evansi*) and dourine (*Trypanosoma equiperdum*) in camels and horses (Giordani et al. 2016). The centenary of the unveiling of suramin’s chemical structure provides the opportunity to review the little-known histories surrounding the secret development and the political instrumentalization of the drug.

## Discovery and proof of efficacy of suramin

**Fig. 1:**
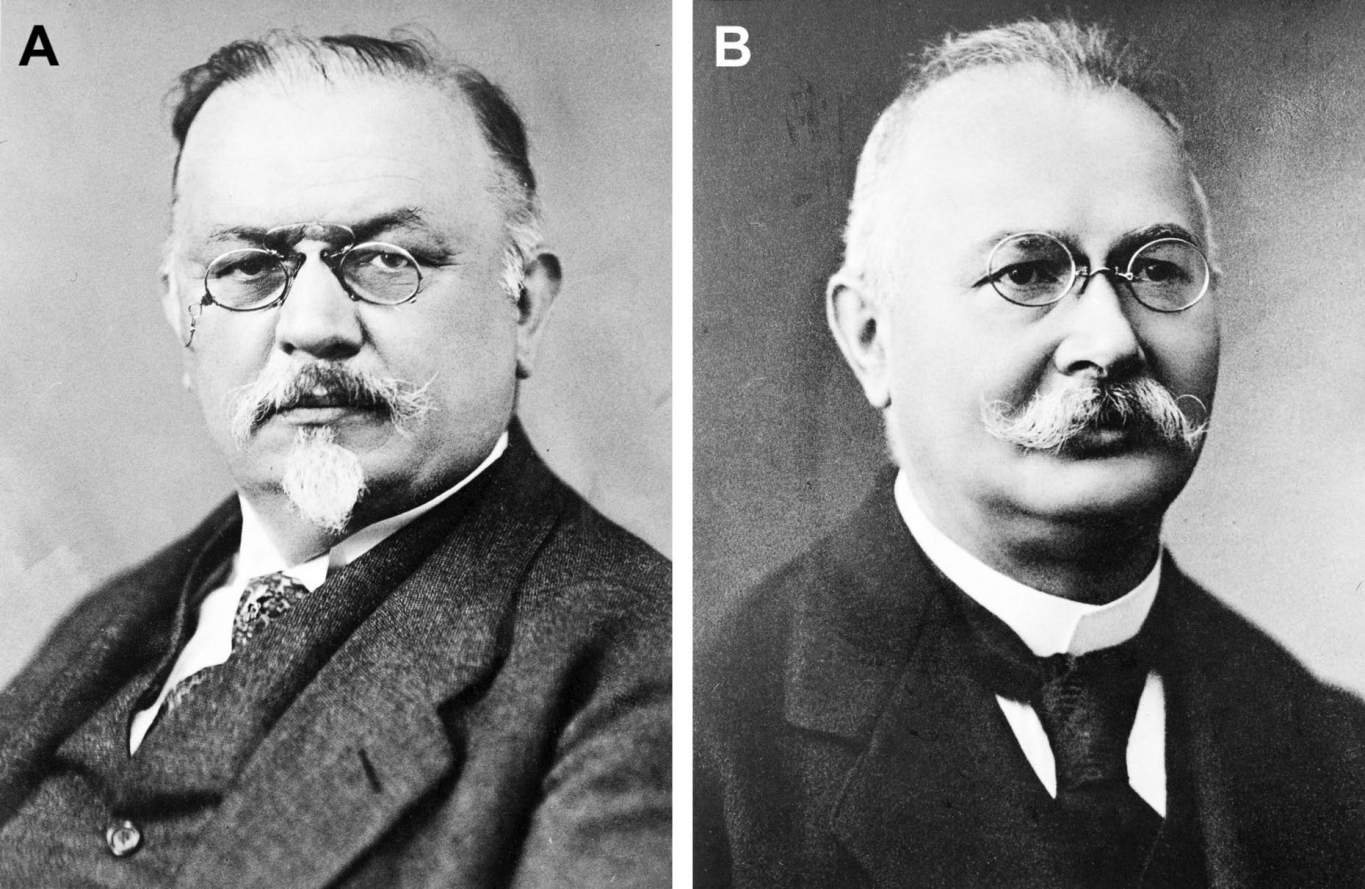
Portraits of Oskar Dressel (**A**) and Richard Kothe (**B**). Dressel and Kothe synthesized the anti-sleeping sickness drug suramin. Wellcome Collection. Attribution 4.0 International (CC BY 4.0). Links: **A**, https://wellcomecollection.org/works/pkvppgr9;**B**, https://wellcomecollection.org/works/nu8ztnrq

**Fig. 2:**
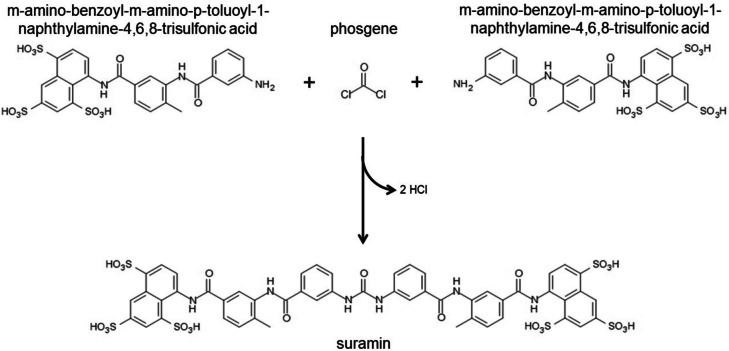
Synthesis and structure of suramin. Despite being very effective in killing bloodstream forms of *T. brucei*, the drug does not follow any of the currently accepted criteria for drug-likeness. The molecular weight of suramin is >500 g/mol (1297 g/mol), the drug has more than 5 hydrogen bond donors (12) and more than 10 hydrogen bond acceptors (23), and suramin’s partition coefficient Log *P* is not within the range of −0.4 to +5.6 (−2.33)

As the original company name suggests (Farbenfabriken means dyestuff factories), Bayer was initially an enterprise developing and manufacturing synthetic dyes for the textile industry (Bayer 2023). Based on Bayer’s reputation as one of the world’s leaders in the synthesis of dyes and on earlier work by Paul Ehrlich (1854–1915) showing that certain dyes are toxic to trypanosomes (Sneader 2005), the French researchers Maurice Nicolle (1862–1932) and Felix Mesnil (1868–1938) approached the Bayer company and ordered the synthesis of benzopurpurine compounds, a class of red/blue azo dyes usually used for dying cotton (Dressel 1941; Dressel and Oesper 1961). Thereupon, the scientific laboratory director at Bayer’s research group in Elberfeld (Wuppertal), Bernhard Heymann (1861–1933), asked the chemists Oskar Dressel (1865–1941) (Fig. 1) and Richard Kothe (1863–925) (Fig. 1) in 1906 to synthesize the requested benzopurpurine dyes (Dressel 1941; Dressel and Oesper 1961; Travis 1991). One compound named Trypan Blue displayed strong trypanocidal activity and eliminated trypanosomes from the blood of infected animals (Steverding 2010). The research group at Bayer also became interested in testing the benzopurpurine derivatives, and Wilhelm Roehl (1881–1929), a former assistant of Paul Ehrlich who joined the Bayer research group at Elberfeld in 1905, was asked to carry out chemotherapeutic studies (Dressel 1941; Dressel and Oesper 1961; Steverding 2010). However, Roehl complained that the dyes were of no value as they were staining the animals bluish or reddish and therefore not useable in patients (Dressel 1941; Dressel and Oesper 1961; Steverding 2010). He asked for colorless compounds that he could test for trypanocidal activity (Dressel 1941; Dressel and Oesper 1961). Roehl’s request was not unrealistic, since the researchers had come across less colored or colorless dyes. In addition to preparing benzopurpurine derivatives for Nicolle and Mesnil, Dressel and Kothe decided to synthesize their own compounds (Dressel 1941; Dressel and Oesper 1961). However, they opted for a different class of molecules and chose to explore the field of urea chemistry (Dressel 1941; Dressel and Oesper 1961). It was not long before Kothe discovered that 2-amino-5-naphthol-7-sulfonic acid (J acid) could be easily converted into a urea compound by reaction with phosgene (Dressel 1941; Dressel and Oesper 1961). The J acid could be readily extended by introducing amino-benzoyl nuclei, and soon, it was discovered that compounds with four amino-benzoyl groups had promising but insufficient activity to kill trypanosomes in experimental animals (Dressel 1941; Dressel and Oesper 1961). Although this early progress was encouraging and the first patents were filed, the supervising authorities became doubtful about the chemotherapeutic potential of dyes after several years of further efforts without a breakthrough (Dressel 1941; Dressel and Oesper 1961). Due to Heymann’s support and influence, permission was granted to Dressler and Kothe to continue with the experiments at Bayer (Dressel 1941; Dressel and Oesper 1961). Finally, in the autumn of 1917 and after synthesizing and testing over 1000 naphthalene ureas, they found the magic bullet: the urea obtained by the reaction of m-amino-benzoyl-m-amino-p-toluoyl-1-naphthylamine-4,6,8-trisulfonic acid with phosgene (Fig. 2) (Dressel 1941; Dressel and Oesper 1961). The compound was initially named Bayer 205.

Soon after Bayer 205 was shown to be very effective in eliminating trypanosomes from the blood of infected animals, the first therapeutic attempt was carried out in 1921 on two returning Europeans who contracted sleeping sickness during their stay in southern Africa (Mayer 1922; Jacobi 2010; Madeja and Schroeder 2020). As both had little chance of survival, they were treated with Bayer 205. After a few injections of the compound, both patients recovered and traveled back to Africa (Mayer 1922; Jacobi 2010). Although Bayer only partially documented the therapeutic success, it was clear that Bayer 205 was effective in humans and larger field trials were planned. In the same year, the German government in collaboration with Bayer organized a sleeping sickness clinical trial expedition to the British colony of Rhodesia. Under the leadership of the pharmacologist Friedrich Karl Kleine (1869–1951), the expedition traveled to South Africa on the 15th of October 1921 and in November 1921 set off from Cape Town to Rhodesia (Eckart 1990; Madeja and Schroeder 2020). Although the British actively supported the expedition, the major problem was the low number of sleeping sickness patients on which suramin could be tested (Eckart 1990). However, the promising initial results prompted the chief physician of the Katanga Province of Belgian Kongo to invite the expedition in 1923 to treat the numerous sleeping sickness patients present there^2^ (Eckart 1990; Madeja and Schroeder 2020). Encouraged by the great success of these expeditions, it was decided to place Bayer 205 on the market in 1923 under the connotative name “Germanin” at the suggestion of the Foreign Office (Eckart 1990; Madeja and Schroeder 2020).

## Political instrumentalization and publication of the suramin formula

Renaming Bayer 205 with the patriotic name “Germanin” was an attempt by the Germans to politically instrumentalize the drug with a view to repossessing former colonies in Africa (Jacobi 2010). In accordance with the Treaty of Versailles that formally ended World War One, Germany had to renounce sovereignty over all its overseas colonies (Encyclopædia Britannica 2023). Initial animal experiments with Bayer 205 at the Reichsgesundheitsamt (Imperial Health Office) in Berlin and the Institut für Schiffs- und Tropenhygiene^3^ (Institute for Marine and Tropical Hygiene) in Hamburg were carried out under heightened secrecy (Jacobi 2010), and the subsequent clinical trial expeditions were planned with top secrecy so that the discovery of Bayer 205 was not revealed (Jacobi 2010). In addition, private and governmental sectors requested that Bayer should not reveal the “Key to Africa” (Pope 1924). Bayer therefore did not disclose the formula of the drug. Also, for fear of foreign competitors, the public was not informed about the discovery and trials of the new drug until the successful completion of the expeditions in 1923 (Jacobi 2010). From then on, an increasing number of articles were published in newspapers by German colonial societies demanding the political instrumentalization of the drug (Jacobi 2010). In particular, a lurid article by the colonial enthusiast Hans Zache (1869–1930) suggesting the exchange of Germanin for the allegedly unjust loss of the former German colonies caused outrage among the Entente nations when the article made its way into the Canadian newspaper “Montreal Daily Star” in January 1924 (Jacobi 2010). Moreover, Great Britain and France rejected outright Germany’s suggestion that it would disclose the formula of Germanin in exchange for its former colonies (Madeja and Schroeder 2020). To avoid further diplomatic damage, the German Consulate General in Montreal together with the German Foreign Ministry released a disclaimer stating that Germany would no longer request the return of its former colonies (Jacobi 2010). Thus, the publication of Zache’s article destroyed Germany’s hope for a moral revision of its colonial past.

**Fig. 3:**
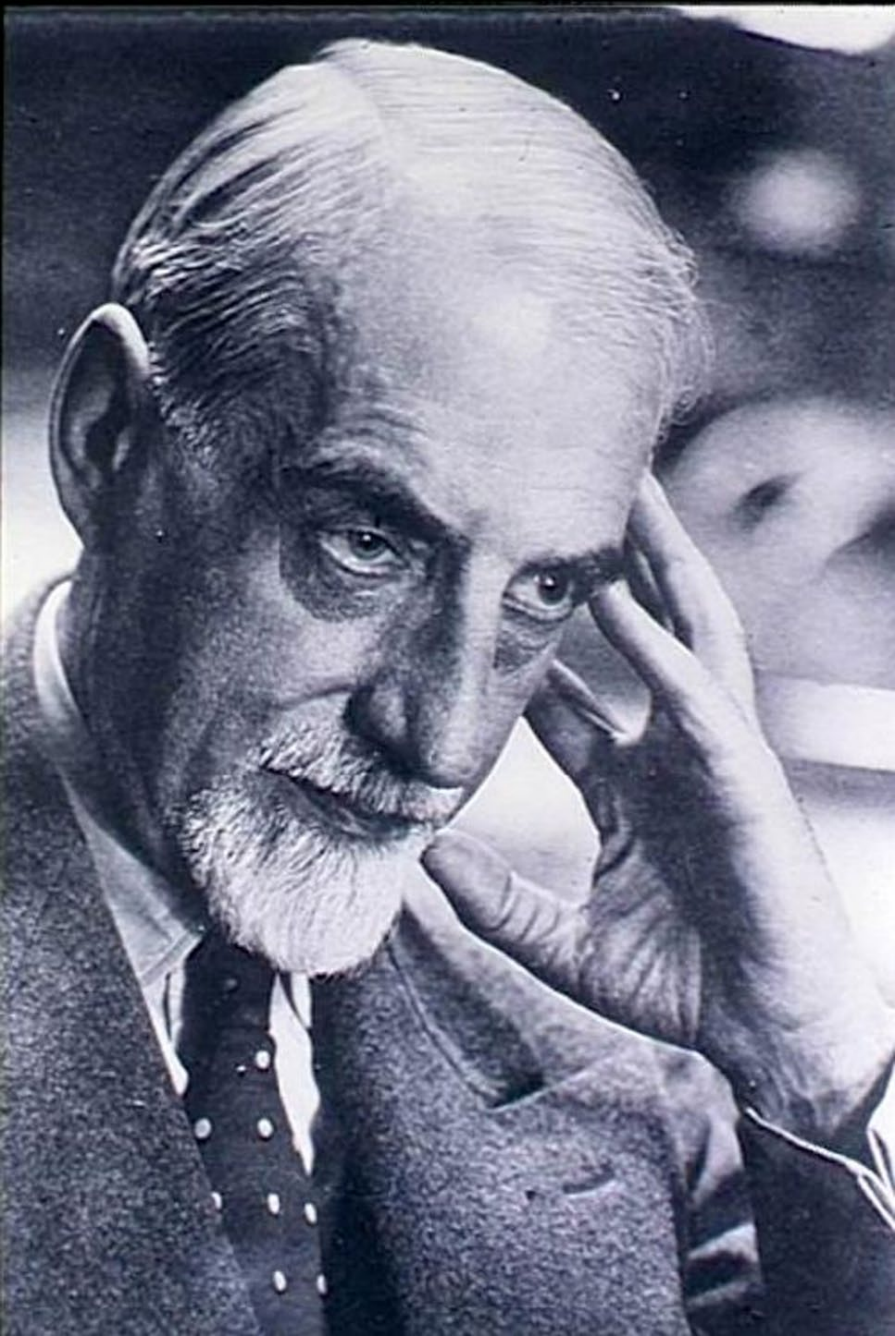
Portrait of Ernest Fourneau who uncovered the chemical structure of suramin. Wikimedia Commons. Creative Commons CC0 License. Link: http://gallica.bnf.fr/ark:/12148/btv1b32000224/f41.item

Also, any anticipated commercial success with Germanin was shattered in 1924 when the French pharmacist Ernest Fourneau (1872–1949) (Fig. 3) published the chemical formula of Bayer 205 (Fourneau et al. 1924). After having reviewed the 17 patents Bayer had filed for naphthalene sulfonic acid ureas with trypanocidal activity, Fourneau deduced that Bayer 205 could have only one of 25 possible structures (Fourneau et al. 1924; Sneader 2005). Subsequently, he synthesized several of the compounds and had their trypanocidal effect tested in infected mice (Fourneau et al. 1924; Sneader 2005). By comparing the antitrypanosomal properties of the compounds with the trypanocidal activity of Bayer 205, he concluded that Germanin must be the symmetrical urea of m-amino-benzoyl-m-amino-p-toluoyl-1-naphthylamine-4,6,8-trisulfonic acid (Fourneau et al. 1924). Because the formula of Bayer 205 had not been previously published, Bayer could not sue Fourneau for infringing its patents (Sneader 2005). The subsequent agreement between Bayer and the French company Poulenc-Frères to jointly market the drug dampened the remaining financial hopes for Germanin (Jacobi 2010). In 1928, Bayer finally confirmed that Bayer 205 was identical to Fourneau’s structure (Sneader 2005) and as of 1944, the drug was officially termed suramin (Ewins 1944), although the exact etiology of the revised name is unclear.

## Colonial revisionism during national socialism

Those who thought that the disclosure of suramin’s formula and Germany’s renunciation of its former colonies would end the discussion of colonial revisionism in Germany would be proven wrong. When the Nazis came to power in 1933, Germanin was used to illustrate the success of German research and to again promote colonial-revisionist efforts. For example, no opportunity was missed to discredit British and French sleeping sickness control measures and the view was expressed that Germany had demonstrated considerable proficiency in fighting tropical diseases (Jacobi 2010). However, in contrast to the previous plans of the Weimar Republic to colonize Africa, the Nazis intended to exploit Africa’s commodity markets (Jacobi 2010). Nevertheless, the story of Germanin’s discovery was made the subject of literature and films in which the alleged unjust loss of Germany’s former colonies was condemned.

In 1938, the ophthalmologist and Nazi writer Hellmuth Unger (1891–1953) published the book *Germanin - Geschichte einer deutschen Großtat* (Eng. transl.: Germanin - Story of a German feat) (Unger 1938), which tells the perceived tragedy that the loss of German colonies hampered Germanin’s potentially transformative effects on those living in these regions. The reader is repeatedly reminded that Germany could have legitimately stopped the testing of Germanin but instead continued to be committed to the community of nations in times of national degradation and humiliation (Jacobi 2010). Although the book contains historical facts about the discovery of Germanin as well as detailed portraits of renowned sleeping sickness researchers (Robert Koch (1843–1910), David Bruce (1855–1931), Robert Michael Forde (1861–1948), and Joseph Everett Dutton (1874–1905)), the 1921/23 sleeping sickness expedition leader Friedrich Karl Kleine, and the founders of the Bayer company (Friedrich Bayer (1825–1880) and Johann Friedrich Weskott (1821–1876)), the main focus of the work was the reiteration of old colonial-revisionist arguments.

The political instrumentalization of Germanin peaked in the filming of Unger’s book. The 92-min-long feature film entitled *Germanin - Die Geschichte einer kolonialen Tat* (Eng. trans.: Germanin - The story of a colonial deed) and directed by Max W. Kimmich (1893–1980) was premiered at the Hamburg UFA-Palast on the 15th of May 1943 (Eckart 1990; Jacobi 2010)]. The plot of the film revolves around the 1921/23 African sleeping sickness expeditions carried out to test Germanin under field conditions (for a brief synopsis of the storyline see Box 1). Although the main characters and the storyline of the film were loosely based on this event, many details did not correspond with historical facts. For instance, the anti-British propaganda of the film painted the image that the British obstructed the German expedition (Eckart 1990; Jacobi 2010). However, personal notes of the expedition leader Friedrich Karl Kleine suggested the opposite (Jacobi 2010). Since the exchange of Germanin for the former colonies was no longer a possibility, the film was purely made for propaganda purposes.

**Table:** 

Box 1. Synopsis of the film *Germanin - Die Geschichte einer kolonialen Tat*. In Africa, the renowned German Professor Achenbach together with his young female assistant Anna Meinhart are conducting research to find a remedy against the deadly sleeping sickness. (The role of Professor Achenbach is inspired by Friedrich Karl Kleine who led the 1921/23 expeditions evaluating Germanin’s efficacy under field conditions while that of the assistant Meinhart is based on Kleine’s laboratory technician and later wife Hanna Ockermann (Eckart 1990).) A breakthrough is imminent when Professor Achenbach learns that the First World War has erupted in Europe and that the British are also fighting the Germans in Africa. The laboratory of the Professor is raided and burnt down. However, with the help of his friend Dr. Hofer, he can rescue important research results. (The role of the animal trapper Dr. Hofer is largely fictitious but later as Achenbach’s coworker resembles Kleins’s medical assistant Walter O. Fischer (Eckart 1990).) Back in Germany, Achenbach continues his research and discovers the groundbreaking sleeping sickness drug “Germanin.” He wants to return to Africa where the new drug is needed badly. After tough negotiations, the British allow him to come back to Africa, but when he and his colleagues arrive there, they are constantly harassed and obstructed. Displeased with Achenbach’s treatment success in the colonies, the British commander Colonel Crosby orders the destruction of all Germanin stocks. Soon afterwards Achenbach gets infected with sleeping sickness. A last vial of Germanin that by chance had escaped destruction could save the Professor. Just at the moment when he wants to inject himself, Colonel Crosby appears, also infected with sleeping sickness. In exchange for a promise that Crosby will allow forest clearing to destroy the breeding places of the tsetse flies, Achenbach saves the British Colonel by giving him the last vial, while he valiantly dies.	

## Suramin, a drug with numerous potential applications

Even before the formula of suramin was disclosed, the effect of the drug against other diseases was tested. Already in November/December 1922, several individuals suffering from kala-azar were treated with suramin although the drug turned out to be ineffective in curing the patients (Napier 1923). Since then, suramin has been trialed in the treatment of many different diseases ranging from parasitoses and viral infections to cancer, arthritis, and autism (Wiedemar et al. 2020). Based on the discovery that suramin inhibits the reverse transcriptase of retroviruses (De Clercq 1979), the effects of the drug on the human immunodeficiency virus (HIV) were tested. Although it was shown that suramin could reduce the viral burden in some AIDS patients, no improvement in their immunological status or clinical symptoms was observed (Cheson et al. 1987). More recently, the drug has been shown to be effective in inhibiting Sars-CoV-2 infection in cell culture (Saldago-Benvindo et al. 2020). For a short period of time, suramin was used in the treatment of river blindness, a helminthic disease caused by the nematode *Onchocerca volvulus* (Wiedemar et al. 2020). However, suramin is no longer in routine use to treat onchocerciasis and has been replaced by the orally administrable drug ivermectin (Wiedemar et al. 2020). Thus, East African sleeping sickness and the animal diseases surra and dourine remain the only conditions that presently are still treated with suramin.

## Conclusion

The political instrumentalization of suramin and the non-disclosure of its formula had far-reaching consequences for both the German government at the time and the Bayer company. The government of the Weimar Republic had to acknowledge once and for all the loss of its former colonies. The publication of suramin’s formula destroyed Bayer’s hope for financial profit from the drug. Thus, the non-patenting and non-disclosure of the structure of suramin backfired for Bayer. Because of this, the disclosure of the structures of drugs subsequently became standard practice in pharmaceutical patents (Sneader 2005).

## Data Availability

The authors declare that data supporting the findings of this study are available within the article.

## Footnotes

1 While *T. b. gambiense* is the causative agent of West African sleeping sickness which is an anthroponotic and a more chronic disease, *T. b. rhodesiense* is responsible for the East African form of the infection which is a zoonotic and a more acute disease (Steverding 2017).

2 In contrast to previous clinical trials with arsenic drugs conducted by German colonial physicians in German colonies before 1914, this time, there was no need to forcefully treat patients as many came voluntarily. Nevertheless, the human testing of suramin remains ethically questionable (Eckart 1990).

3 The Institut für Schiffs- und Tropenhygiene was renamed Bernhard-Nocht-Institut für Schiffs- und Tropenkrankheiten in 1942 on the occasion of the 85th birthday of Bernhard Nocht, the founder of the institution. In 1990, the institution was renamed Bernhard-Nocht-Institut für Tropenmedizin (BNITM 2023).
